# Food Waste to Wellness: A Grape Pomace Blend in Gastric Cancer Prevention

**DOI:** 10.1002/fsn3.70350

**Published:** 2025-05-30

**Authors:** Matteo Micucci, Michela Battistelli, Sabrina Burattini, Michele Mari, Michele Retini, Riham Osman, Ilaria Versari, Anna Bartoletti Stella, Federico Gianfanti, Jude Okeke Udodinma, Francesco Onesimo, Serena Riela, Matteo Canale, Antonio Palumbo Piccionello, Irene Faenza, Sara Salucci

**Affiliations:** ^1^ Department of Biomolecular Sciences University of Urbino Carlo Bo Urbino Italy; ^2^ Department of Biomedical and NeuroMotor Sciences University of Bologna Bologna Italy; ^3^ Department of Medical and Surgical Sciences University of Bologna Bologna Italy; ^4^ Dipartimento di Scienze Chimiche University of Catania Catania Italy; ^5^ Biosciences Laboratory IRCCS Istituto Romagnolo per Lo Studio Dei Tumori (IRST) “Dino Amadori” Italy; ^6^ Dipartimento di Scienze e Tecnologie Biologiche Chimiche e Farmaceutiche (STEBICEF) Università Degli Studi di Palermo Palermo Italy

**Keywords:** circular economy, food by‐products, functional foods, nutraceuticals, polyphenols, stomach cancer

## Abstract

This study investigates the chemopreventive potential of a grape pomace extract (GPE) derived from Verdicchio grapes, aligning with circular economy principles to repurpose winery waste into a nutraceutical targeting gastric cancer prevention. Soxhlet extraction yielded a bioactive‐rich extract. Comprehensive chemical characterization via HPLC/ESI/Q‐TOF identified 39 metabolites spanning key chemical classes. Anthocyanins were predominant, with malvidin glucoside (12,546 mg/kg DM; 36.3%), malvidin coumaroyl glucoside (9941 mg/kg DM; 28.8%), and malvidin acetylglucoside (7189 mg/kg DM; 20.8%) as the most abundant compounds. Carboxylic acids included tartaric, malic, isocitric, aconitic, and succinic acids, while lipid molecules, such as phytosphingosine and stearic acid, and amino acids like proline, valine, leucine, and phenylalanine further enriched the extract's chemical heterogeneity.

Biological evaluations revealed GPE's selective cytotoxicity against AGS (IC_50_: 13.64 μg/mL) and KATO III (IC_50_: 7.11 μg/mL) gastric cancer cells, sparing GES‐1 cells. Mechanistically, GPE‐induced apoptosis through caspase‐3 activation and mitochondrial dysfunction, as evidenced by inner membrane disruption and cardiolipin peroxidation. TEM and CLSM morphological analyses revealed hallmark apoptotic features, including chromatin condensation, micronuclei formation, and apoptotic bodies. Additionally, GPE impaired autophagy, marked by Beclin‐1 downregulation and LC3B‐II upregulation. The accumulation of degradative vacuoles indicated disrupted autophagosome clearance, shifting autophagy from a survival mechanism to a cell death‐promoting pathway. These findings highlight GPE's dual impact on apoptosis and autophagy in gastric cancer cells, underscoring its potential as a dietary intervention for gastric cancer prevention.

## Graphical abstract

Grape pomace was used to set up a phytocomplex studied for gastric cancer prevention. HPLC/MS analyses revealed that this mixture is a source of many polyphenols and amino acids. This novel nutraceutical (GPE) selectively targets cancer cells, sparing healthy ones. Molecular mechanisms confirmed that GPE acts, inducing apoptosis and autophagy. All merged data suggested GPE application in circular economy and cancer prevention.

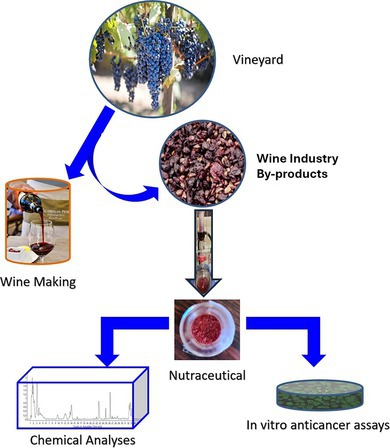

## 1 Introduction

Gastric carcinoma (GC) represents a major challenge in global health, marked by striking variations in incidence across the continents. The multifactorial nature of GC arises from a combination of genetic, microbial, environmental, and lifestyle factors. Dietary habits, microbial influences, environmental factors, and lifestyle choices contribute to GC's multifactorial etiology (Thrift et al. 2023). While surgical and chemotherapeutic approaches remain the primary strategies for GC management, integrating preventive strategies is crucial. Epidemiological studies have extensively documented the significant impact of dietary habits on gastric cancer incidence, with plant‐based diets consistently correlating with reduced gastric cancer risk (Bai et al. 2023). Polyphenols, flavonoids, and other bioactive compounds from plant‐based sources have been shown to influence multiple molecular pathways associated with gastric carcinogenesis. Experimental evidence further supports the efficacy of these bioactives in inhibiting proliferation and inducing apoptosis in GC cells. However, despite the growing scientific interest in these compounds, there is still a need to explore underutilized plant‐derived sources that could serve as potential nutraceuticals for cancer prevention. Nutraceutical research focuses on identifying molecules that modulate gastric cancer onset and progression, targeting critical biological mechanisms, including apoptosis induction, inflammation attenuation, and oxidative stress suppression. Phytochemicals, phytocomplexes, and traditional Chinese medicine (TCM) formulations have demonstrated in vitro efficacy against multiple cancer types, including gastric cancer (Micucci et al. 2024). In addition, some food components have been reported to sensitize tumor cells to chemotherapy by modulating autophagic processes (El‐Ganainy et al. 2023; Sritharan et al. 2022). These substances play a dual role in gastric cancer progression, modulating tumor growth, proliferation, and metastasis (Ting et al. 2021; Xu et al. 2020). Autophagy, a key biological process, removes dysfunctional proteins and organelles, recycling them into simple molecules. This self‐degradative mechanism sustains cellular homeostasis and metabolic adaptation (Ahn et al. 2007). However, while autophagy can support cancer cell survival under stress conditions, excessive activation has been associated with autophagic cell death, particularly in response to phytochemical treatment (Sritharan et al. 2022). Many food‐derived substances were shown to affect autophagic mechanisms, shifting the balance toward apoptotic events (Musial et al. 2021). Given their role in modulating multiple oncogenic pathways, these bioactive compounds hold promise for the development of functional ingredients with potential applications in gastric cancer prevention. Molecules found in wine and grape‐derived foods demonstrated antiproliferative and proapoptotic effects across various cancer models, interacting with multiple signaling pathways (Ferreira‐Santos et al. 2024; Otun et al. 2024). Several studies have investigated the anticancer effects of isolated grape‐derived compounds, such as resveratrol (Zhou et al. 2022), malvidin, and glycosidic derivatives, proanthocyanidins (Kim et al. 2024), demonstrating their role in apoptosis induction and oxidative stress modulation. Additionally, grape seed and skin extracts have been tested in colorectal cancer and breast cancer (Lee et al. 2023), with promising results in inhibiting tumor progression. However, while the bioactivity of individual polyphenols and grape extracts has been widely explored, no studies have specifically investigated the effect of grape pomace extract (GPE) on gastric cancer. Given that gastric cancer remains a major global health challenge, our study aims to fill this gap by evaluating the selective cytotoxicity of GPE against gastric cancer cells and elucidating its molecular mechanisms. Without proper management, this material is typically discarded, leading to environmental concerns such as greenhouse gas emissions from decomposition. Despite its high content of bioactive compounds, grape pomace remains underexploited, presenting an opportunity for its valorization as a nutraceutical ingredient. Addressing this issue through valorization strategies aligns with sustainability principles and enhances the circular economy approach (Muhlack et al. 2018). In this context, our study was designed to explore the potential of grape pomace as a novel functional ingredient with in vitro anticancer properties.

This study embraces circular economy principles by transforming grape pomace, a by‐product from cultivars grown in the Marche region, into a nutraceutical blend (Mallamaci et al. 2021). Through chemical characterization and in vitro evaluation, GPE demonstrated its potential as a functional ingredient with selective anticancer activity against GC cells.

This dual‐purpose approach not only highlights the health‐promoting potential of GPE but also favors sustainable practices by valorizing winemaking by‐products.

## 2 Materials and Methods

### 2.1 Grape Pomace Extraction and Chemical Characterization

#### 2.1.1 Sample Collection and Preparation

The grape pomace was sourced from a local vinicultural enterprise in the Castelli di Jesi area, located in the Marche region of Italy, and obtained from the Verdicchio cultivar ( *Vitis vinifera* L.). This region is renowned for its high‐quality viticulture, with Verdicchio grapes being particularly valued for their richness in bioactive compounds. This cultivar distinguishes between a protected geographical indication (PGI) and a protected designation of origin (PDO), attesting to its origin authenticity and quality assurance. The cultivation regimen was conducted in strict accordance with the principles of organic agriculture, adhering to comprehensive ecological and agronomic protocols.

#### 2.1.2 Extraction Process

The pomace was carefully cleaned and destemmed before being lyophilized for 72 h using an LIO5P freeze dryer (Pascale, Italy). Extraction was performed using a Soxhlet apparatus (Büchi, Switzerland) with a solvent mixture of 70% ethanol and 30% water, acidified with 3 g/L citric acid (Codex Alimentarius compliant). A total of 20 g of lyophilized pomace was placed in a cellulose thimble and extracted with 180 mL of solvent under continuous reflux at 90°C for 4 h. The Soxhlet system ensured repeated extraction cycles with constant solvent renewal, allowing for dynamic diffusion and progressive solubilization of free and bound polyphenols from the matrix. The condenser coil was continuously cooled using recirculating water at ambient temperature to ensure efficient condensation and solvent recovery throughout the extraction. The process was conducted at 90°C for 4 h, utilizing 20 g of lyophilized pomace and 180 mL of solvent. The resulting extract was dried using a rotary evaporator, yielding a fine powder stored under cryogenic conditions at −18°C. Solvents and reagents, including ethanol (≥ 99.8%) and citric acid (≥ 99.5%), were sourced from Sigma‐Aldrich (St. Louis, MO, USA).

**TABLE 1:** Metabolites identified in GPE using HPLC/MS/ESI/Q‐TOF.

	Compound	Rt (min)	ESI^+^ [M]^+^ or [M + H]^+^ (m/z) (*Exp*.)	ESI^+^ [M]^+^ or [M + H] (m/z) (*Teor*.)	Molecular formula	Metabolites classes
1	Tartaric acid	1.18	151.0217	151.0237	C_4_H_6_O_6_	Carboxylic acids
2	Valinol	1.33	104.1066	104.1070	C_5_H_13_NO	Peptides
3	Proline	1.52	116.0707	116.0706	C_5_H_9_NO_2_	Peptides
4	Valine	1.65	118.0823	118.0862	C_5_H_11_NO_2_	Peptides
5	Leucine	1.78	132.1021	132.1019	C_6_H_13_NO_2_	Peptides
6	Phenylalanine	1.90	166.0844	166.0862	C_9_H_11_NO_2_	Peptides
7	dihomocitric acid	2.22	221.0652	221.0656	C_8_H_12_O_7_	Carboxylic acids
8	Tripeptide	2.73	322.1787	322.1761	C_16_H_23_N_3_O_4_	Peptides
9	Amino‐octanoic acid	2.99	160.1327	160.1332	C_8_H_17_NO_2_	Carboxylic acids
10	5‐Phenylvaleric acid	3.16	179.1162	179.1067	C_11_H_14_O_2_	Carboxylic acids
11	Dihydroresveratrol	3.71	231.1060	231.1016	C_14_H_14_O_3_	Stilbenes
12	Malvidin glucoside	4.44	493.1331	493.1341	C_23_H_25_O_12_	Anthocyanins
13	3,5‐Dicaffeoylquinic acid	9.60	517.1327	517.1340	C_25_H_24_O_12_	Cinnamic acids
14	Peonidin acetylglucoside	12.06	505.1336	505.1341	C_24_H_25_O_12_	Anthocyanins
15	Malvidin acetylglucoside	12.18	535.1441	535.1446	C_25_H_27_O_13_	Anthocyanins
16	Cyanidin coumaroyl glucoside	13.23	595.1432	595.1446	C_30_H_27_O_13_	Anthocyanins
17	Petunidin coumaroylglucoside	13.34	625.1543	625.1552	C_31_H_29_O_14_	Anthocyanins
18	3,5‐Di‐O‐caffeoyl‐4‐O‐coumaroylquinic acid	13.56	663.1699	663.1708	C_34_H_30_O_14_	Cinnamic acids
19	Peonidin coumaroylglucoside	13.96	609.1558	609.1603	C_31_H_29_O_13_	Anthocyanins
20	Malvidin coumaroylglucoside	14.02	639.1675	639.1708	C_32_H_31_O_14_	Anthocyanins
21	Tetradecadienoyl‐homoserine lactone	15.85	308.2179	308.2220	C_18_H_29_NO_3_	Lipids
22	Dehydrophytosphingosine	22.63	316.2824	316.2846	C_18_H_37_NO_3_	Lipids
23	Phytosphingosine	24.84	318.2972	318.3003	C_18_H_39_NO_3_	Lipids
24	Sphinganine	26.20	302.3050	302.3053	C_18_H_39_NO_2_	Lipids
25	Hydroxyicosatrienoic acid Isomer I	32.24	323.2571	323.2581	C_20_H_34_O_3_	Lipids
26	Hydroxyicosatrienoic acid Isomer II	32.56	323.2575	323.2581	C_20_H_34_O_3_	Lipids
27	Hydroxyicosatrienoic acid Isomer III	33.04	323.2570	323.2581	C_20_H_34_O_3_	Lipids

#### 2.1.3 High‐Performance Liquid Chromatography/Mass Spectrometry (HPLC/MS) Analysis

HPLC/MS analysis was performed by adapting previously reported methods (Faddetta et al. 2023; Raimondo et al. 2022). HPLC/MS analysis samples were prepared by dissolving 5 mg of grape pomace in 1 mL of methanol (MeOH). Water and acetonitrile were HPLC/MS grade, and formic acid was of analytical quality. A reversed‐phase Phenomenex Luna C18(2) column (150 × 4.6 mm, particle size 3 μm) with a Phenomenex C18 security guard column (4 × 3 mm) was used. The injection volume was 25 μL. The eluate was monitored with mass total ion count (MS TIC) and UV (530 nm). Mass spectra were obtained on an Agilent 6540 UHD accurate‐mass quadrupole–time‐of‐flight (Q‐TOF) spectrometer equipped with a dual AJS electrospray ionization (ESI) source working in positive or negative mode. Nitrogen N_2_ was used as desolvation gas at 300°C with a flow rate of 8 L min^−1^. The nebulizer was set to 45 psig. The sheath gas temperature was set at 400°C with a flow of 12 L min^−1^. A potential of 3.2 kV was applied to the capillary in positive mode and 2.6 kV in negative mode. The fragment was set to 75 V. MS spectra were recorded in the 100–1000 m/z range. Quality control was performed prior to analysis using mass calibration in the range of 100–3000 Da (Q‐TOF calibration mix) and solvent delay calibration for retention time according to the manufacturer's instructions. An in‐house quality check mix containing known compounds (phenylalanine, saccharose, benzoic acid, and rutin) was injected during the batch of analysis. Mass spectrum data were analyzed for metabolites annotation using MassHunter Qualitative Analysis B.06.00 and the Metabolomics Workbench database [https://www.metabolomicsworkbench.org/]. MS/MS spectra were recorded with a collision energy of 10–40 V. Compounds annotated through MS/MS data are reported in Tables 2 and 3. Other compounds were identified by comparison with commercial analytical standards. Quantitative analyses were performed using malvidin glucoside as a standard for relative quantitative analysis of anthocyanins. A stock solution containing 100 mg/L was prepared in methanol. Other solutions were prepared by successive dilutions with water using the stock solution, with the following concentrations: 0.1, 1, 10, 25, 50, and 100 mg/L. The quantitation was performed based on the extracted ion count (EIC) for the [M] + ion for all anthocyanins.

**TABLE 2:** Metabolites identified using MS/MS experiments in positive mode.

	Compound	Rt (min)	ESI^+^ [M − H]^−^ (m/z) (*Exp*.)	ESI^+^ [M − H]^−^ (m/z) (*Teor*.)	Molecular formula	Metabolites classes
1	Gallic acid	2.02	169.0135	169.0143	C_7_H_6_O_5_	Phenolic acid
2	Aconitic acid	2.12	173.0110	173.0092	C_6_H_6_O_6_	Carboxylic acids
3	isocitric acid	2.16	191.0215	191.0197	C_6_H_8_O_7_	Carboxylic acids
4	Succinic acid	2.17	117.0202	117.0193	C_4_H_6_O_4_	Carboxylic acids
5	Monoethylsuccinate	2.30	145.0517	145.0506	C_6_H_10_O_4_	Carboxylic acids
6	Malic acid	2.42	133.0147	133.0143	C_4_H_6_O_5_	Carboxylic acids
7	Citric acid	2.55	191.0218	191.0197	C_6_H_8_O_7_	Carboxylic acids
8	Caftaric acid	25.42	311.0432	311.0409	C_13_H_12_O_9_	Cinnamic acids
9	Quercetin	27.53	301.0382	301.0384	C_15_H_10_O_7_	Flavonoid
10	Ursolic acid	28.21	455.3561	455.3531	C_30_H_48_O_3_	Triterpenic acid
11	PI (18:4)	29.42	591.2610	591.2576	C_27_H_45_O_12_P	Phospholipids
12	Stearic acid	30.99	283.2665	283.2643	C_18_H_36_O_2_	Fatty acid

*Note:* Metabolites identified using HPLC/MS/ESI/Q‐TOF in positive mode.

**TABLE 3:** Metabolites identified using MS/MS experiments in negative mode. Refers to Table 2 in the main manuscript for numbering.

	mg/Kg DM	Area%
Malvidin glucoside	12,546 ± 21	36.3
Peonidin acetylglucoside	1176 ± 8	3.4
Malvidin acetylglucoside	7189 ± 14	20.8
Cyanidin coumaroyl glucoside	297 ± 11	0.9
Peonidin coumaroylglucoside	2073 ± 5	6.0
Petunidin coumaroylglucoside	1332 ± 12	3.9
Malvidin coumaroylglucoside	9941 ± 23	28.8

*Note:* Metabolites identified using HPLC/MS/ESI/Q‐TOF in negative mode.

### 2.2 Cell Culture and Treatments

The study was performed on three commercial GC cell lines (AGS, KATO III, and SNU‐1) purchased from ATCC (American Type Culture Collection, Manassas, VA, USA). Epithelial gastric cells (GES‐1) were kindly provided by Prof. Mario Dell'Agli, Department of Pharmacological and Biomolecular Sciences “Rodolfo Paoletti,” University of Milan, 20133 Milan, Italy. AGS cells, isolated from a female patient with gastric adenocarcinoma, were grown in DMEM/F12 medium (Gibco, Thermo Fisher Scientific, Waltham, MA, USA) supplemented with penicillin 100 units/mL (Gibco, Thermo Fisher Scientific, Waltham, MA, USA), streptomycin 100 mg/mL (Gibco, Thermo Fisher Scientific, Waltham, MA, USA), L‐glutamine 2 mM (Gibco, Thermo Fisher Scientific, Waltham, MA, USA), and 10% heat‐inactivated fetal bovine serum (FBS, Euroclone Milano, Italy). KATO III cell line has been established in vitro from a pleural effusion of a 55‐year‐old Asian male stomach cancer patient and cultured with Iscove's modified Dulbecco's medium (ATCC) supplemented with 20% FBS. For SNU‐1, isolated from the stomach of a 44‐year‐old Asian male patient with carcinoma, the culture medium was composed of RPMI‐1640 medium (ATCC) supplemented with 10% FBS (Tedaldi et al. 2021). GES‐1 cells, a noncancerous gastric epithelial immortalized cell line, were used as a control. These cells, derived from normal gastric epithelial tissue, were cultured in RPMI‐1640 medium (Gibco, Thermo Fisher Scientific, Waltham, MA, USA) supplemented with 10% FBS, penicillin 100 units/mL, streptomycin 100 mg/mL, and 1% L‐glutamine (Gibco, Thermo Fisher Scientific, Waltham, MA, USA). All cell lines, including GES‐1, were maintained at 37°C with 5% CO_2_ and subcultured weekly. GPE, dissolved in DMSO, was administered to all cell lines. Control wells included untreated cells and cells treated with an equivalent concentration of DMSO as the vehicle control. GPE was added to the culture media at different concentrations (62, 125, and 250 μg/mL) and incubation periods (24, 48, and 72 h). Subsequently, cells underwent viability, proliferation, and ultrastructural assessments.

#### 2.2.1 Trypan Blue (TB) dye Exclusion Assay

Cell viability was assessed using TB exclusion assay. Cells (1.0 × 10^5^ cells/mL) were seeded in multiwell plates, treated with GPE, detached using trypsin, and resuspended in 1× PBS containing 0.5 mM EDTA and 0.2% BSA. 50 μL of the cell suspension was mixed with 50 μL of 0.4% TB, incubated for 5 min at room temperature, and a 10 μL aliquot was loaded onto a hemocytometer. Viable (clear) and dead (blue) cells were counted under a light microscope at 40× magnification. Viability was calculated as follows:

$$\%\text{Viability}=\left(\text{Viable cells}/\text{Total cells}\right)\times 100$$

Measurements were performed with an Invitrogen Countess 3 Automated Cell Counter (Thermo Fisher) using at least three biological replicates per condition.

#### 2.2.2 MTT Assay

The cytotoxicity of GPE was evaluated on gastric cells (KATO III, AGS, GES‐1) using the MTT assay. Cells (200,000/well) were seeded into 96‐well plates and incubated at 37°C, 5% CO_2_ for 24 h. GPE (1–120 μg/mL) was added, ensuring that the DMSO concentration was lower than 0.1% (v/v) to prevent solvent toxicity. Controls included untreated cells and cells treated with DMSO only. After 72 h at 37°C, 20 μL of MTT solution (5 mg/mL) was added to each well and incubated for 4 h. The cells were then washed three times with 1XPBS, and the formazan crystals were dissolved in 100 μL DMSO. Absorbance was measured at 590 nm. Cytotoxicity was calculated from the absorbance values and analyzed using GraphPad Prism v9.0, with an *r* ^*2*^ > 0.5 considered valid.

### 2.3 Ultrastructural Analyses

#### 2.3.1 Scanning Electron Microscopy (SEM)

AGS and KATO III cells were washed and fixed with 2.5% glutaraldehyde in 0.1 M phosphate buffer for 15 min. The cells were then scraped and centrifuged at 300× g for 10 min. The pellets were fixed in 2.5% glutaraldehyde for an additional 30 min. The suspended cells were collected in Eppendorf tubes, centrifuged, and fixed for 45 min in glutaraldehyde. Afterward, the suspended cells were deposited on poly‐L‐lysine–coated coverslips overnight at 4°C. All specimens were postfixed with 1% osmium tetroxide (OsO_4_) in 0.1 M phosphate buffer for 1 h. After alcohol dehydration, the samples were critical point dried, gold sputtered, and observed using an ESEM microscope (UMKC, Kansas City, MO, USA) (Salucci et al. 2017).

#### 2.3.2 Transmission Electron Microscopy (TEM)

AGS, KATO III, and GES‐1 cells were washed and fixed with 2.5% glutaraldehyde in 0.1 M phosphate buffer (pH 7.3) for 15 min. The cells were scraped and centrifuged at 300× g for 10 min. The pellets were fixed in 2.5% glutaraldehyde for an additional 30 min. The suspended cells were collected in Eppendorf tubes, centrifuged, and immediately fixed for 45 min in glutaraldehyde. The samples were postfixed in 1% OsO_4_ for 1 h, alcohol dehydrated, and embedded in Araldite (Battistelli et al. 2015). For ultrastructural analysis, thin sections were stained with UranyLess and lead citrate and analyzed using a Philips CM10 transmission electron microscope (FEI Italia SRL, Milano, Italy).

#### 2.3.3 Confocal Laser Scanning Microscopy (CLSM)

Specimens were observed with a Leica TCS‐SP5 CLSM connected to a DMI 6000 CS Inverted Microscope (Leica Microsystems CMS GmbH; FITC and PI [propidium iodide] excitation were at 488 and 500 nm, respectively, and their emission signals were detected at 525 and 617 nm). CLSM images are presented as maximum intensity projections or single‐plane images.

##### 2.3.3.1 Acridine Orange (AO) and PI Nuclear Staining

AO and PI fluorescence probes were used to distinguish between early apoptosis, late apoptosis, and necrosis (Salucci et al. 2014). AO diffuses into viable and early apoptotic cells, emitting green fluorescence, while PI binds DNA in membrane‐compromised cells, emitting red fluorescence. Early apoptotic cells show a bright‐green nucleus with chromatin condensation or micronuclei. Late apoptotic cells emit orange fluorescence from AO and PI binding, while necrotic cells show red fluorescence from PI. AGS and KATO III cells were cultured on coverslips, and SNU‐1 cells were seeded on polylysinated coverslips. All samples were fixed in 4% paraformaldehyde (PBS, pH 7.4) for 30 min and washed twice in PBS. Cells were pretreated with RNase (10 μg/mL, Life Technologies) for 30 min, then stained with AO and PI (1 μg/mL each, diluted in PBS) at room temperature in the dark for 10 min.

##### 2.3.3.2 Mitochondrial Staining

For mitochondrial analysis, a fluorescent probe known as 10‐N nonyl‐acridine orange (NAO) has been used to stain the inner mitochondrial membrane and detect the potential peroxidation events. Therefore, cells grown as described above have been treated with 50 nM NAO for 10 min in PBS. After PBS washing, slides were mounted with an antifading medium (Vectashield, Vector Labs) (Salucci et al. 2016).

### 2.4 Western Blotting (WB)

Cells were lysed in RIPA buffer with Halt protease and phosphatase inhibitors (Thermo Fisher, USA) by vortexing on ice for 30 min. Lysates were cleared by centrifugation (14,000× g, 10 min, 4°C), and protein concentrations were determined using a BCA protein assay kit (Thermo Fisher, USA). Proteins were resolved on 4%–20% SDS‐PAGE gels and transferred to nitrocellulose membranes via semidry blotting. Membranes were blocked in PBS with 5% BSA (60 min, RT) and incubated overnight at 4°C with primary antibodies: Beclin‐1 (Cat #3495, CST), caspase‐3 (Cat #9661, CST), LC3B (Cat #3868, CST), and β‐actin (Cat #sc‐1616, SCBT), all diluted at 1:1000. After four washes in PBS‐Tween 20, membranes were incubated with peroxidase‐conjugated secondary antibody (1:10,000, 1 h, RT) and washed. Bands were visualized using ECL and imaged with a ChemiDoc Imager (Bio‐Rad, USA).

### 2.5 Statistical Analysis

Statistical analyses were performed to assess the efficacy of GPE on the viability of gastric cell lines (AGS, KATO III, SNU‐1, and GES‐1). The TB dye exclusion assay data representing cell viability as a percentage relative to the untreated control groups were subjected to statistical examination. MTT assay data are presented as mean ± standard deviation (SD) derived from at least three independent experiments. All statistical analyses were conducted using GraphPad Prism 9.0 (GraphPad Software, San Diego, CA, USA). Three independent experiments were conducted for statistical analysis, and the results were averaged. The normality of data distributions was assessed using the Shapiro–Wilk test. Assumption checks for the two‐way ANOVA were performed to ensure normality and homogeneity of variances. Levene's test was used to confirm the homogeneity of variances. Additionally, Tukey's test was used for post hoc comparisons with corrections applied for multiple comparisons to maintain the validity of the statistical inferences. Missing data were excluded from the analysis.

Results were considered statistically significant at *p* < 0.05. The significance levels were indicated: * for *p* < 0.05 and ** for *p* < 0.01.

## 3 Results

In this study, grape pomace from the Verdicchio cultivar underwent Soxhlet extraction, chosen for its ability to recover bioactive compounds from complex matrices efficiently. The exhaustive extraction facilitated a comprehensive phytochemical profile for chemical characterization. Seventy percent ethanol, a GRAS solvent, was employed for its proven efficacy in extracting phenolics, such as anthocyanins and other polyphenols, supporting its suitability for food and nutraceutical applications.

### 3.1 Chemical Characterization

**FIGURE 1:**
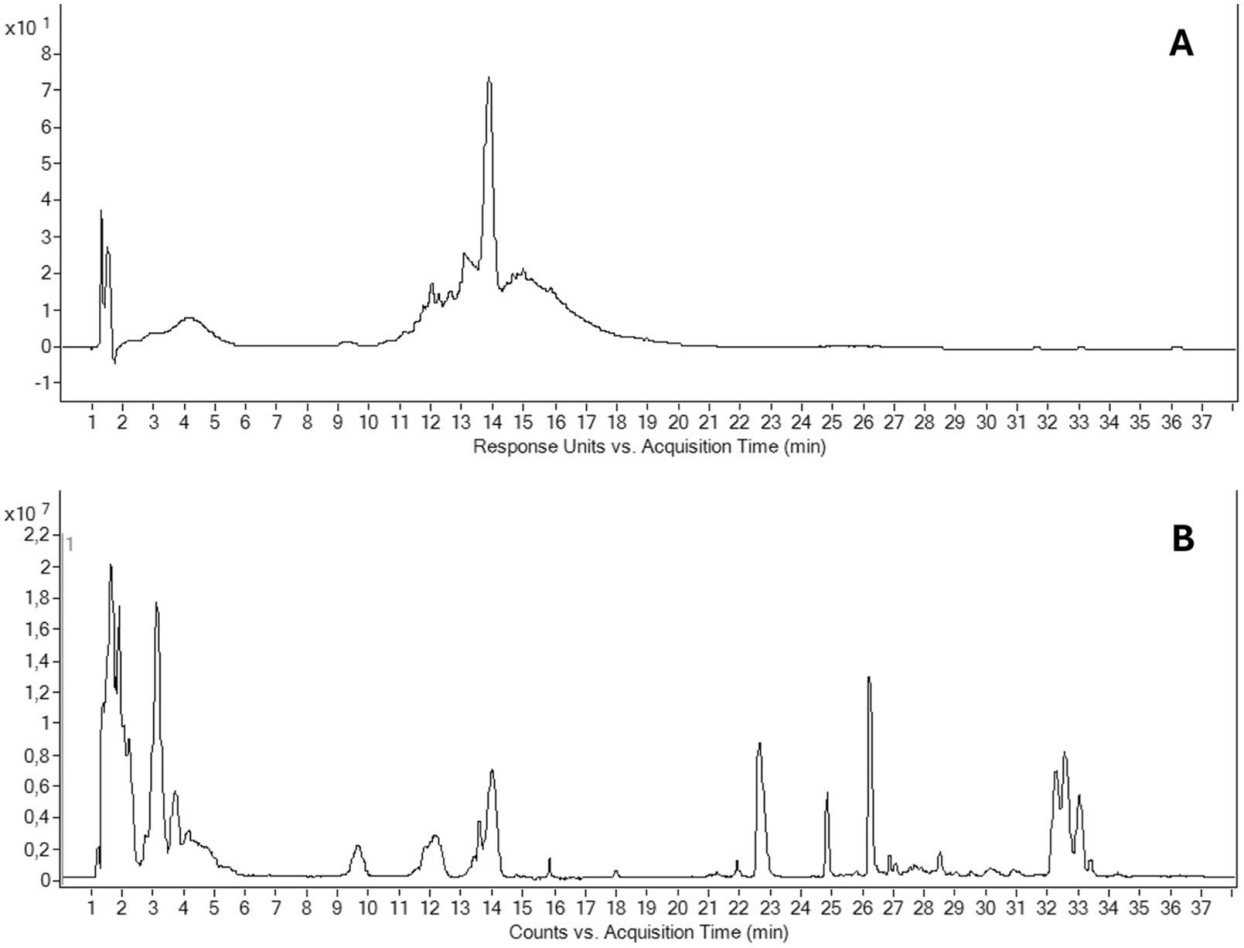
Representative HPLC traces of grape pomace. UV trace at 530 nm (up) and total ion counts (TIC) (down). Detailed results can be found in Tables 1 and 2.

HPLC/ESI/Q‐TOF analysis revealed 39 metabolites in GPE Figure 1, including 27 in positive mode (Table 1) and 12 in negative mode (Tables 2 and 3). To assess the composition of UrbX, we conducted an HPLC/ESI/Q‐TOF analysis in positive and negative modes. As depicted in Figure 1, this analysis allowed us to identify and quantify 39 distinct metabolites within Grape Pomace, 27 in positive mode (Table 1) and 12 in negative mode.

Anthocyanins dominated the profile, with malvidin glucoside as the most abundant (12,546 mg/kg DM; 36.3% of total anthocyanins), followed by malvidin coumaroyl glucoside (28.8%) and malvidin acetylglucoside (20.8%). Other anthocyanins included peonidin coumaroyl glucoside (6.0%), petunidin coumaroyl glucoside (3.9%), peonidin acetylglucoside (3.4%), and cyanidin coumaroyl glucoside (0.9%) (Table 4).

**TABLE 4:** Quantitative distribution of anthocyanins in the grape pomace.

	Compound	Molecular formula	MS/MS peaks
10	Ursolic acid	C_30_H_48_O_3_	248
11	PI (18:4)	C_27_H_45_O_12_P	429, 275

Carboxylic acids identified were tartaric, dihomocitric, amino‐octanoic, 5‐phenylvaleric, aconitic, isocitric, succinic, acetohydroxybutyrate, malic, and citric acids.

Cinnamic acids included 3,5‐dicaffeoylquinic acid, 3,5‐di‐O‐caffeoyl‐4‐O‐coumaroylquinic acid, and caftaric acid. Additional compounds comprised gallic acid, quercetin, dihydroresveratrol, ursolic acid, lipids (e.g., phytosphingosine and stearic acid), amino acids (valinol, proline, valine, leucine, and phenylalanine), and one tripeptide. Quantification confirmed malvidin glucoside's dominance among anthocyanins, supported by EIC analysis. These findings highlight the diverse phytochemical composition of GPE.

### 3.2 Cell Viability TB and MTT Assays

**FIGURE 2:**
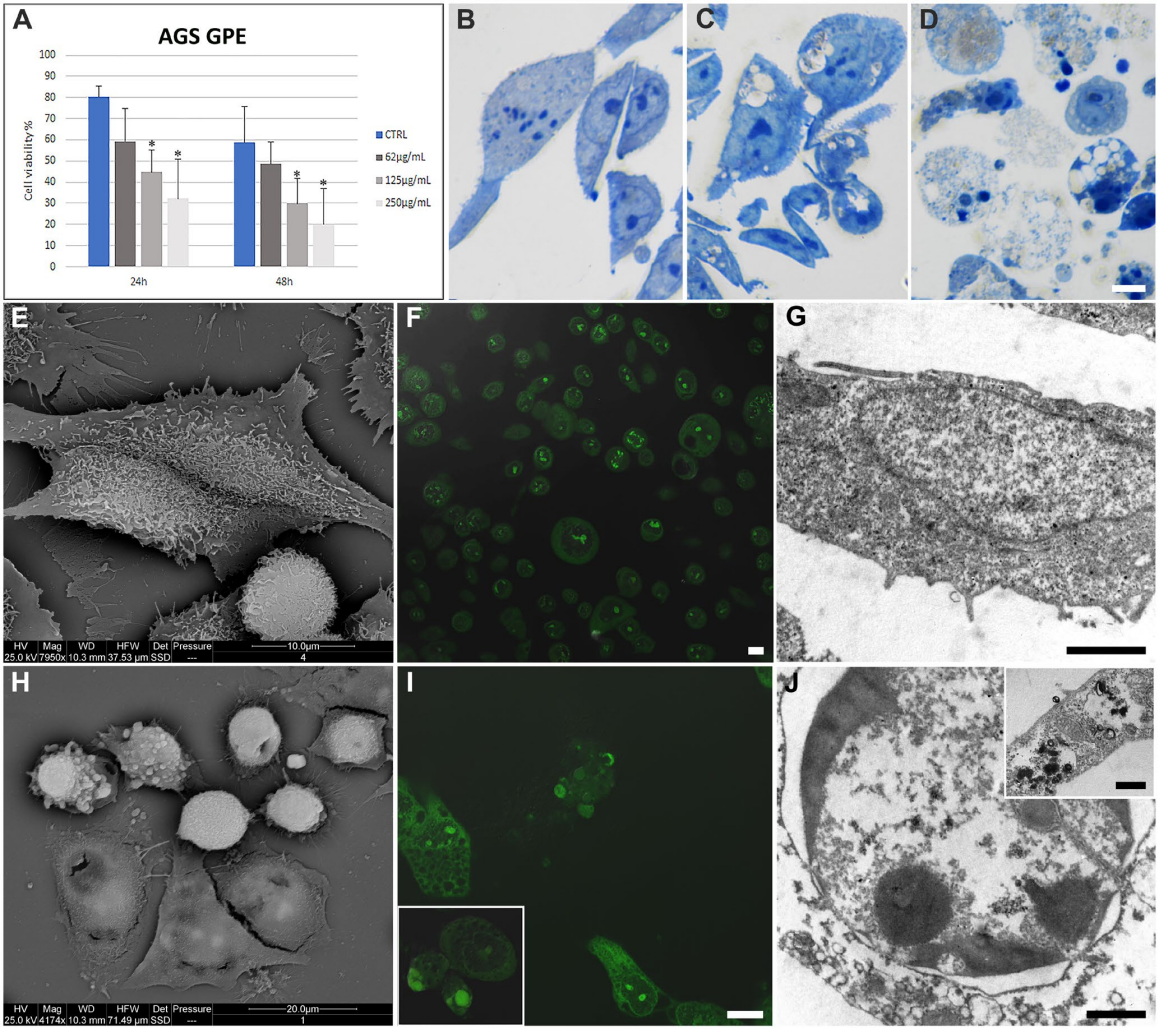
Cell viability percentage was evaluated through TB assay in AGS control and GPE‐treated cells at 24 and 48 h (A). Optical observation of control (B), adherent (C), and suspended (D) and GPE‐treated cells. ESEM observations (E, H) of control (E) and 125 μg/mL GPE‐treated (H) cells. Fluorescent micrographs of AO and PI were stained in control (F) and treated (I), and AGS cell lines were inset (I). TEM analysis of control (G) and treated AGS cells (J), inset (J). B–D, F, I, inset I, Bar = 10 μm; G, L, Bar = 1 μm; inset L, Bar = 0.5 μm. * for CTRL vs. 125 μg/mL and CTRL vs. 250 μg/mL at 24 h of treatment.

GPE treatment reduces AGS cell viability in a dose and time‐dependent manner, as demonstrated by TB assay (Figure 2A). This behavior occurs in a concentration range of 62–250 μg/mL. The 125 μg/mL concentration was the better option, as it induced a higher apoptotic rate than the highest concentration, which resulted in necrotic events. We excluded the 250 μg/mL GPE concentration because it induces necrosis that can lead to a high proinflammatory effect.

**FIGURE 3:**
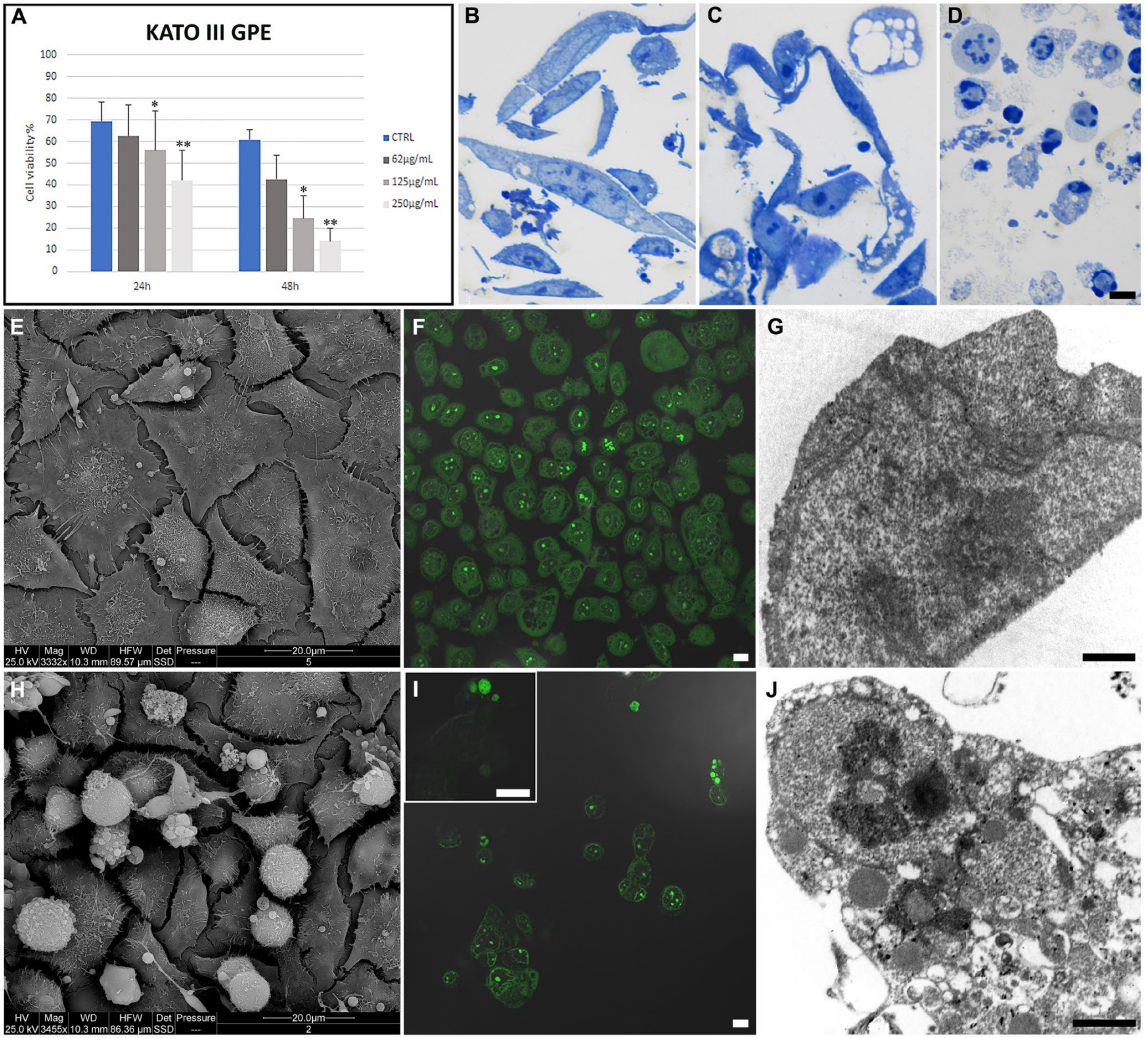
Cell viability percentage was evaluated through TB assay in KATO III control GPE‐treated cells at 24 and 48 h (A). Optical observation of control (B), adherent (C), and suspended (D) GPE‐treated cells. ESEM observations (E, H) of control (E) and 125 μg/mL GPE‐treated (H) cells. Fluorescent micrographs of AO‐ and PI‐stained control (F) and treated I, inset (I) KATO III cell lines. TEM analysis of control (G) and treated cells (J). B–D, F, I, inset (I), Bar = 10 μm; G, L, bar = 1 μm. * CTRL vs. 125 μg/mL; **CTRL vs. 250 μg/mL after 24 h of treatment. CTRL corresponds to untreated cells.

TB assay demonstrated that GPE treatment reduces KATO III (Figure 3A) cell viability in a dose‐ and time‐dependent manner with a concentration range of 62 to 250 μg/mL.

GPE treatment indicates a trend toward reduced viability in SNU‐1 cells, as shown in Figure 3A. Still, this reduction is not statistically significant, indicating a weak effect of GPE treatment against these types of cancer cells. Cells treated with an equivalent concentration of DMSO show viability comparable to that of the control condition (data not shown). Therefore, data presented in the graphs are relative to DMSO‐treated cells (considered as control).

**FIGURE 4:**
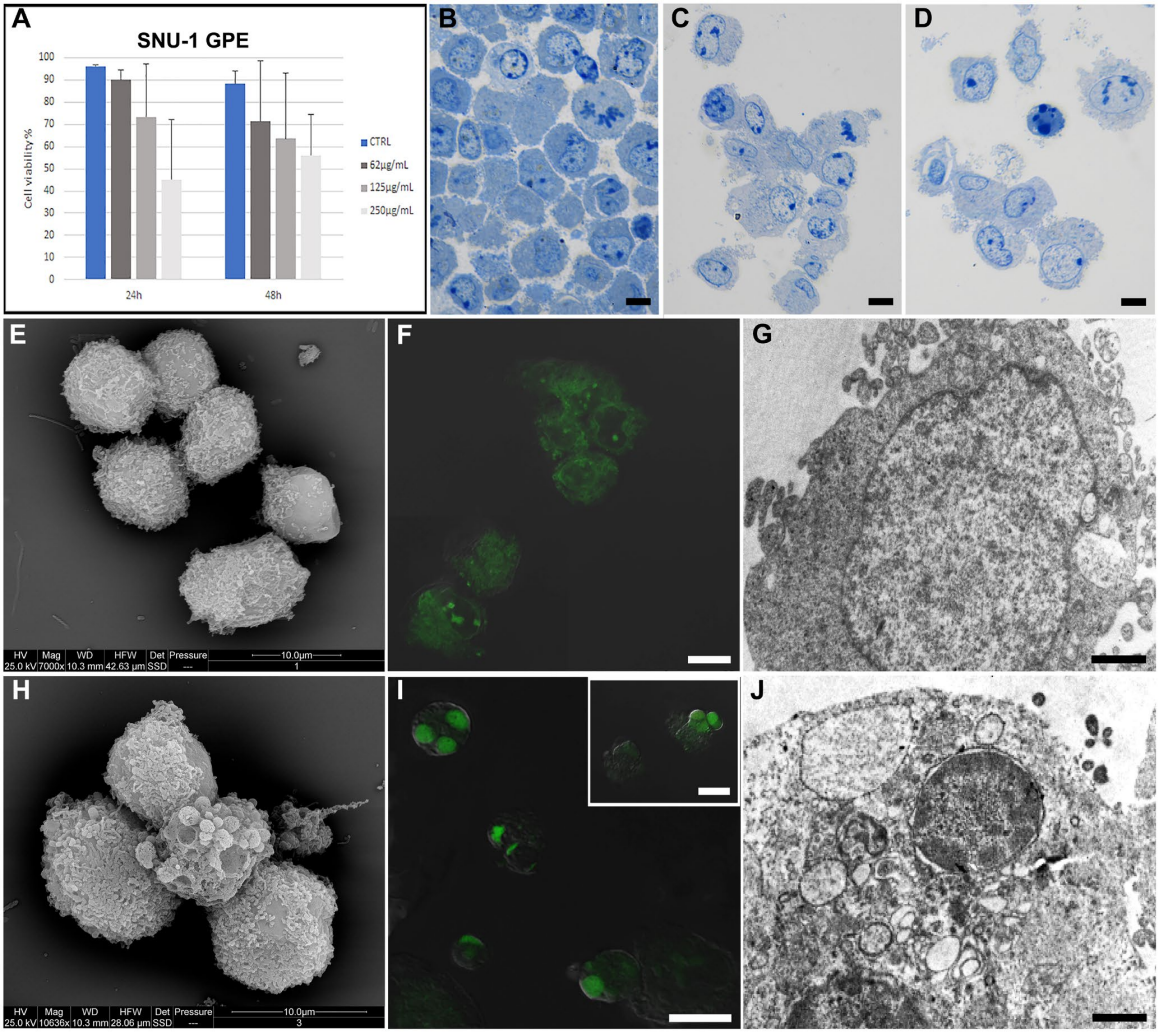
Cell viability percentage evaluated through TB assay in control cells and 62, 125, 250 μg/mL GPE‐treated SNU‐1 cells at 24 and 48 h (A). Optical observation of control (B), adherent (C), and suspended (D) GPE‐treated cells. ESEM observations (E–H) of control (E) and 125 μg/mL GPE‐treated (H) cells. Fluorescent micrographs of AO‐ and PI‐stained control (F) and treated (I, inset (I)) SNU‐1 cell lines. TEM analysis of control (G) and treated cells (J). (B–D, F, I, inset (I)) Bar = 10 μm; (G, L) Bar = 1 μm.

**FIGURE 5:**
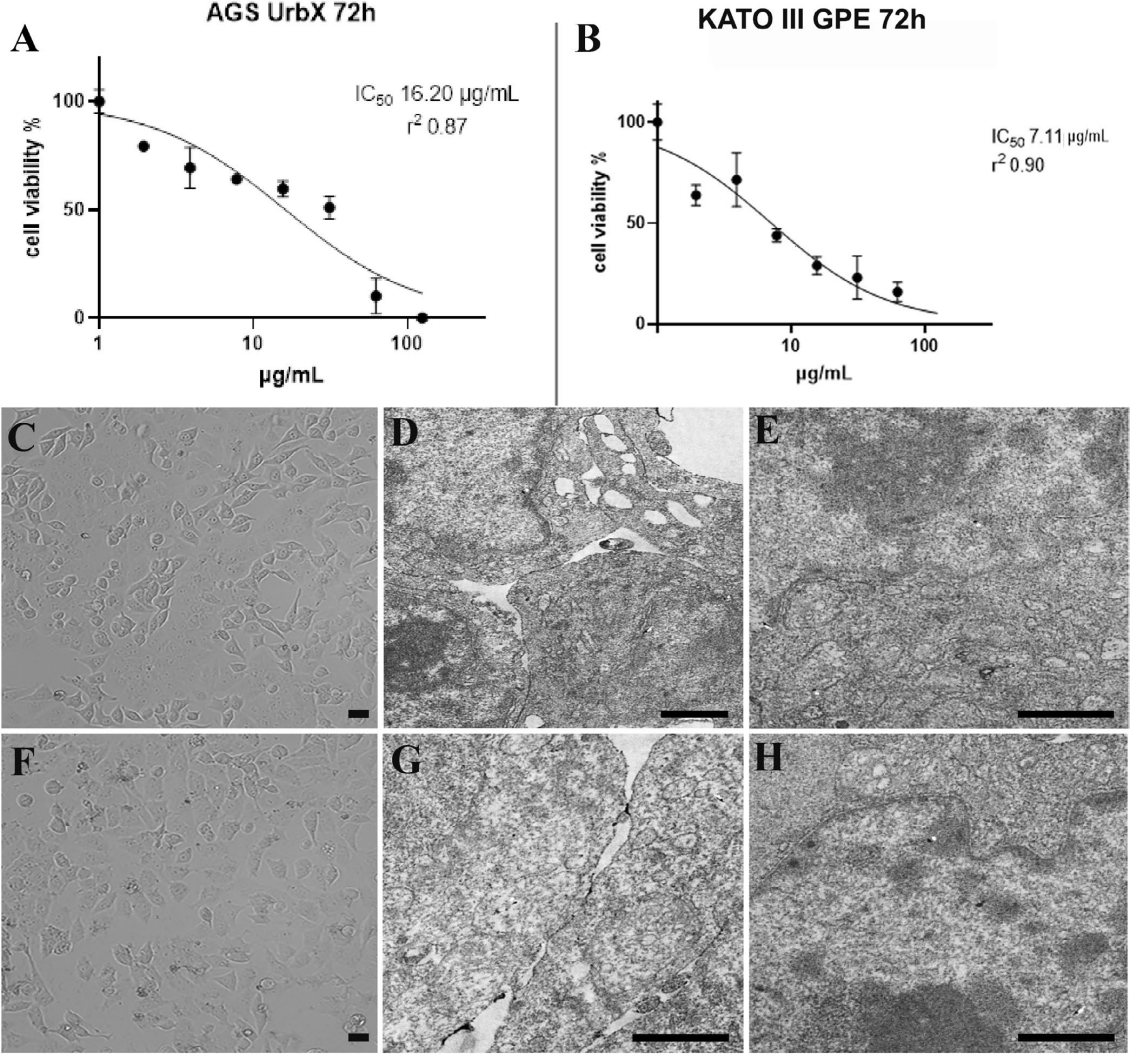
MTT of AGS (A) and KATO III (B). Reverted microscope (C, F) and TEM analyses (D, E, G, H) of control (C–E) and GPE‐treated (F–H) GES‐1 cells. C, F, Bar = 10 μm; D, E, G, H Bar = 1 μm.

The effect of GPE on cell viability was further assessed using the MTT assay after 72 h of treatment. The results show a clear dose‐dependent decline in AGS cell viability, with a significant reduction as the concentration of UrbX increases, yielding an IC50 value of 13.64 μg/mL (Figure 5A). A similar pattern was observed in KATO III cells, where prolonged exposure to GPE after 72 h resulted in significantly higher cytotoxicity, evidenced by a lower IC50 value of 7.11 μg/mL (Figure 5B). This finding suggests that extended exposure to GPE markedly enhances its cytotoxic effects on both cellular lines (Figure 5B).

### 3.3 Morphological Analyses (SEM, TEM, and CLSM)

Control AGS cells exhibited an elongated or polygonal shape (Figure 2B,E), characterized by well‐conserved nuclei, cytoplasmic organelles, and continuous nuclear and cellular membranes (Figure 2F,G). GPE‐treated cells showed progressive damage (Figure 2C,D). A significant decrease in cell confluence, with cell detachment and the appearance of empty spaces, correlated with cell rounding and blebbing (Figure 2H). Some cells presented altered mitochondria, autophagic vacuoles, and intact nucleus and plasma membrane (Figure 2C, inset J). Others became rounded and presented apoptotic nuclear features, such as chromatin condensation and micronuclei presence, which can be observed at LM (Figure 2D,I), CLSM (Figure 2I) and TEM (Figure 2J). AO/PI staining allows the discrimination of the type of death. Most cells are in early apoptosis, as indicated by bright fluorescence spots (Figure 2I and inset I).

Control KATO III cells (Figure 3B,E–G) maintained their ultrastructural integrity regarding cellular membranes and nuclei. Cells appeared mostly polygonal (Figure 3E), and close cell contacts frequently appeared, as confirmed by numerous pseudopodia on the cell surface.

Treatment with GPE led to consistent cell damage (Figure 3C,D,H–J) with diffuse cytoplasmic vacuolization. Cell monolayer showed a significant decrease in cell confluence; rounding and blebbing cells appeared, and some of them were detached from the substrate (Figure 3H). A condensed chromatin area inside the nucleus (Figure 3D) and altered mitochondria and autophagic vacuoles (Figure 3J) could be observed.

This behavior has been confirmed after CLSM observations, too. AO/PI staining revealed the presence of micronuclei (Figure 3I, inset I,J) in cells exposed to GPE, suggesting a consistent apoptosis induction, which is absent in the control condition (Figure 3F).

Control SNU‐1 cells displayed a rounded shape and a preserved morphology (Figure 4B,E–G). After GPE exposure, some cells appeared resistant to the treatment (Figure 4C). In contrast, others showed cytoplasmic vacuoles of varying sizes (Figure 4D), and a certain number of apoptotic cells were revealed by SEM (Figure 4H), CLSM (Figure 4I, inset I), and TEM (Figure 4J) observations.

To evaluate GPE's effect on noncancer cells, we performed the GPE treatment of GES‐1, a noncancerous gastric epithelial immortalized cell line. Reverted microscope (Figure 5C,F) and TEM analysis (Figure 5D–H) show a similar morphology in control (Figure 5C–E) and treated cells (Figure 5F–H), evidencing that GPE acts selectively against gastric cancer cells.

### 3.4 Mitochondrial Staining and Western Blot Analysis

**FIGURE 6:**
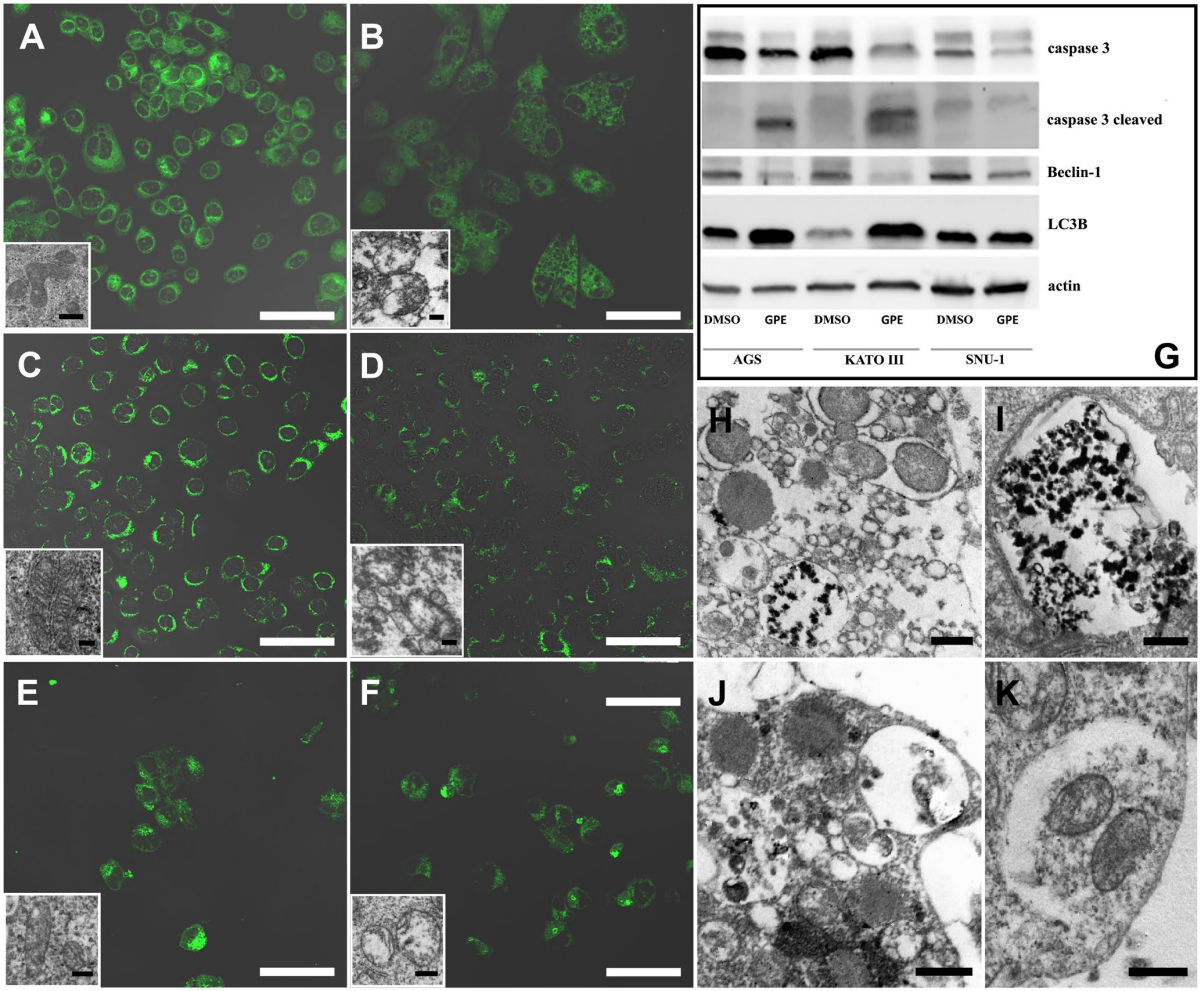
Fluorescent micrographs of NAO‐stained AGS (A, B), KATO III (C, D), and SNU‐1 (E, F) control (A, C, E) and treated (B, D, F) cell lines. Western blot analysis of caspase 3, cleaved caspase 3, Beclin‐1, LC3B, and actin of AGS, KATO III, and SNU‐1 cell lines (G). TEM evaluation of mitochondrial morphological changes in GPE‐treated cells (inset B, D, F) compared to control ones (inset A–inset E) and TEM analyses of autophagic vacuoles in AGS (H, I), KATO III (J), and SNU‐1 (K) 125 μg/mL GPE‐treated cells. A–F, Bar = 50 μm; inset A–inset F, Bar = 1 μm; H, L, M, Bar = 0.5 μm.

We have assessed the presence of peroxidation events to evaluate the integrity of the mitochondrial inner membrane. NAO staining appears more intense in control cells (Figure 6A,C,E) compared to the treated ones (Figure 6B,D,F), as demonstrated by the mitochondria morphology observed using TEM. Cardiolipin peroxidation events can be mostly observed in AGS and KATO III (Figure 6B,D) cells after GPE treatment. At the same time, only a slight fluorescence reduction can be appreciable in SNU‐1‐treated samples (Figure 6F).

Moreover, WB analyses confirmed data obtained from morphofunctional investigations. Apoptotic cell death appeared to be an active process in all cell models, as demonstrated by caspase‐3 reduction and increased expression of cleaved caspase‐3 (Figure 6G). The latter, when activated, is responsible for the morphological changes described above.

Moreover, GPE‐treated samples showed a reduction of Beclin‐1 and an increase of LC3B‐II expression, which suggested the activation of autophagy (Figure 6G). This process has also been described through morphological observations, which revealed many autophagosomes and autophagolysosomes in AGS (Figure 6H,I) and KATO III (Figure 6J) cellular models. In contrast, their quantity decreased in SNU‐1 cells (Figure 6K).

## 4 Discussion

Gastric cancer is one of the leading causes of cancer‐related deaths worldwide. According to the most recent data, in 2020, there were approximately 1 million new cases of gastric cancer and around 769,000 deaths due to this disease (Guan et al. 2023). The current treatments for gastric cancer include surgery, radiation therapy, and chemotherapy, often used in combination. In recent years, targeted therapies and immunotherapies have also shown promise in treating gastric cancer, particularly in patients with advanced disease (Shih et al. 2005). However, the prognosis for patients with gastric cancer depends on the stage of the disease at the time of diagnosis as well as the response to therapy. Therefore, effective preventive measures are warranted.

A preventive approach to gastric cancer could involve substances capable of acting on several molecular targets, affecting the biological networks responsible for gastric cancer onset and progression.

Recently, phytocomplexes and different food compounds such as anthocyanidin, anthocyanins, malvidin, resveratrol, and capable of modulating multiple signaling pathways, resulting in chemopreventive and proapoptotic effects, have been proven as potential preventive measures against gastric cancers (Li et al. 2022; Liu et al. 2023; Micucci et al. 2023; Su et al. 2022; Sun et al. 2023).

In the present study, we focused on a food waste product rich in those molecules endowed with anticancer properties. In this context, we set up a hydroalcoholic extract from Urbino grape pomace that was chemically and biologically investigated.

Chemical characterization revealed a wide variety of compounds in the extract, including carboxylic acids, cinnamic acids, phenolic acids, flavonoids, stilbenes, triterpenic acids, lipids, amino acids, and peptides, all present in lower quantities. A significant proportion of anthocyanins, however, was identified and quantified due to their well‐documented antitumor properties (Shih et al. 2005; Lu et al. 2015). The extraction method adopted in this study was specifically developed to achieve repeated and exhaustive recovery of polyphenolic compounds from grape pomace. Although alternative techniques such as ultrasound‐assisted extraction (UAE), microwave‐assisted extraction (MAE), and maceration have demonstrated efficiency in specific contexts, Soxhlet extraction was selected based on its ability to ensure continuous solvent renewal and a stable diffusion gradient across extraction cycles. Literature comparisons (Brazinha et al. 2014) support that hydroalcoholic Soxhlet extraction, especially when paired with mild acidification, ensures optimal recovery of both free and bound phenolic compounds from complex plant matrices such as grape residues. The 70:30 ethanol/water mixture acidified with 3 g/L citric acid enhances phenolic solubility and helps stabilize anthocyanins. This methodological choice thus reflects a balance between the reproducibility, scalability, and chemical richness of the extract, making it suitable for nutraceutical investigation.

Recent studies have validated the scientific rationale for using citric acid to optimize the extraction of anthocyanins and phenolic acids from grape pomace and similar plant matrices: Yang & Kilmartin (Yang et al. 2025) showed that ethanol acidified with citric acid achieves near‐equivalent anthocyanin yields to HCl, offering a safer and sustainable alternative. Coelho et al. (Coelho et al. 2021) confirmed its effectiveness in food‐grade solvent systems. Kavela et al. (Kavela et al. 2023) demonstrated citric acid's superiority over formic acid. Souza et al. (Galvão et al. 2020) quantified yield improvements due to anthocyanin stabilization at low pH. Chen et al. (Chen et al. 2019) reported enhanced thermal and photostability. Castilla‐Ramírez et al. (Castilla‐Ramírez et al. 2025) confirmed higher pigment retention at high temperatures. These findings support citric acid as a scientifically grounded, food‐grade additive that improves extraction efficiency, safety, and stability.

Moreover, the phytochemical composition of GPEs varies significantly depending on the cultivar and extraction method. The Verdicchio extract exhibited specific anthocyanins, particularly malvidin‐3‐O‐glucoside (12.546 mg/kg) and malvidin‐3‐(6‐O‐coumaroyl)hexoside (28.8 mg/kg), whereas Montepulciano contained malvidin‐3‐O‐acetylhexoside (1.45–1.34 mg/g) and malvidin‐3‐(6‐O‐coumaroyl)hexoside (1.32–1.19 mg/g) (Ferrara et al. 2025). These differences may stem from both cultivar‐specific composition and extraction methodologies, as Soxhlet extraction in Verdicchio likely enhanced anthocyanin recovery, whereas UAE and MAE in Montepulciano favored the extraction of other phenolic compounds (Marinaccio et al. 2025).

Similarly, the Verdicchio extract differed from Ruby Cabernet, which contained a total anthocyanin content of 85.2 mg/g (maceration) and 88.8 mg/g (Soxhlet). The Ruby Cabernet extract also exhibited higher levels of total polyphenols (431.3–224.0 mg/g), flavonoids (146.8–127.2 mg/g), and condensed tannins (31.0–14.4 mg/g), which were not reported in Verdicchio. These variations may be attributed to the methanol‐based extraction in Ruby Cabernet, which enhanced polyphenol recovery. In contrast, ethanol–water Soxhlet extraction in Verdicchio likely preserved anthocyanin stability [García‐Becerra et al. 2016]. These findings highlight the importance of both grape variety and extraction conditions in determining the functional properties of GPEs.

To better contextualize the potency of our Verdicchio GPE, we compared its antiproliferative profile with that of similar extracts reported in the literature. As shown in Table 5, our extract demonstrated notably lower IC_50_ values in AGS and KATO III gastric cancer cells, suggesting enhanced efficacy. The comparative analysis also considers exposure time and mechanistic pathways to strengthen the relevance of our findings.

**TABLE 5:** Comparative IC_50_ values of grape‐derived extracts across different cell lines.

Study	Grapes type	Cell line	IC_50_ (μg/mL)	Exposure time (h)	Molecular mechanisms	Notes
Mišković Špoljarić, K. et al. doi: 10.1186/s12906‐023‐03852‐w	Hydroalcoholic Cabernet Sauvignon extract fermented by white‐rot fungi.	Caco‐2, SW620	910 (Caco‐2), 980 (SW620)	48	Not reported.	The reported IC_50_ values refer to the growth inhibitory concentration leading to a 50% cell growth reduction (GI_50_).
De Sales, N.F.F., et al. doi: 10.3390/molecules23030611	Acidified hydroalcoholic Pinot Noir extract.	HepG2	192	24	Apoptosis.	The number of late apoptotic cells (Annexin^+^/PI^+^) increased after 24 h of GPE treatment.
Luo, J. et al. doi: 10.1080/16546628.2017.1412795	Ultrasonic extraction of Muscadine grape after homogenization in an acetone/methanol/water/formic acid solution (40:40:20:0.1, v/v/v/v).	MDA‐MB‐231	12.9	24	Apoptosis (caspase activation). Cell cycle arrest (cyclin A ↓, p21 ↑).	The crude extract was fractionated. The IC_50_ values refer to F3.
Loizzo, M.R. et al. doi: 10.1016/j.fct.2019.03.007	Ultrasound‐assisted extraction of Greco Nero leaves in a hydroalcoholic solution.	A549, MDA‐MB‐231	96.4 (A549), 28.4 (MDA‐MB‐231)	48	Not reported.	Other five Italian grapevine leaf extracts were tested. Greco Nero had the lowest IC_50_.
Our article	Acidified hydroalcoholic Verdicchio grape pomace extract.	AGS, KATO III	13.64 (AGS), 7.11 (KATO III)	72	Apoptosis (caspase activation).	GPE was selective against gastric cancer cells, sparing healthy cells

*Note:* The table summarizes the antiproliferative effects (IC_50_ values) of various grape pomace extracts, grape types, and cancer cell models from recent literature, along with available exposure times and mechanistic insights. Our Verdicchio GPE exhibits a strong and selective activity toward gastric cancer cell lines (AGS and KATO III).

Our morphofunctional and molecular analyses highlight the potential effect of GPE as a chemopreventive agent in AGS and KATO III gastric cancer cell lines. No toxicity has been evidenced in SNU1 gastric cancer cells, suggesting that intracellular apoptotic pathways activated by GPE are cell‐type dependent.

GPE effects on epithelial gastric cells GES‐1 were also investigated.

The blend selectively targets AGS and KATO III cells without affecting GES‐1 cells. This selective cytotoxicity toward cancer cells can be attributed to several factors: Cancer cells exhibit higher baseline reactive oxygen species (ROS) levels than normal cells, making them more vulnerable to phytochemicals like curcumin and resveratrol, which, in certain conditions, elevate ROS levels, inducing cell death. Normal cells with lower ROS baselines are less impacted (Sun et al. 2023). Cancer cells' reliance on mitochondrial metabolism further sensitizes them to phytochemicals, which disrupt mitochondrial function, releasing proapoptotic factors like cytochrome c and initiating apoptosis. GPE aligns with these mechanisms, impairing mitochondrial integrity in AGS and KATO III cells and promoting apoptosis through oxidative stress and mitochondrial disruption.

This selective anticancer activity of GPE mirrors the effects observed with other phytochemicals.

Anthocyanins and their glycosylated derivatives exhibit selective cytotoxic effects on cancer cells by inhibiting cell proliferation, inducing apoptosis, blocking the cell cycle, reducing inflammation and oxidative damage, and suppressing migration and invasion through modulation of pathways such as PI3K/AKT, MAPK, and MMP (Lu et al. 2015). Malvidin‐3‐glucoside, found in GPE at 12546 ± 21 mg/Kg, selectively induces apoptosis in cancer cells by increasing Bax and decreasing Bcl‐2 levels, disrupting the balance of proapoptotic and antiapoptotic proteins, and promoting programmed cell death while sparing normal cells (De Arruda Nascimento et al. 2022; Merecz‐Sadowska et al. 2023; Rahman et al. 2021). Additionally, peonidin‐3‐glucoside has demonstrated selective antitumor effects on HER2‐positive breast cancer cells by inhibiting cell proliferation and inducing apoptosis through the modulation of HER2, AKT, and MAPK signaling pathways, effectively reducing tumor growth without significant toxicity to normal cells (Liu et al. 2013).

The extract also contains phytosphingosine, which plays a well‐documented role in cancer cell regulation through multiple mechanisms, including mitochondria‐mediated apoptosis, ROS accumulation, inhibition of epithelial–mesenchymal transition (EMT), and metabolic disruption. Specifically, in our study model, this compound may contribute to apoptosis by increasing Bax/Bcl‐2 ratio, triggering mitochondrial cytochrome c release, and activating caspase‐9 and caspase‐3. Additionally, its known ability to inhibit PI3K/Akt and EGFR/JAK1/STAT3 pathways suggests a broader role in disrupting oncogenic signaling. As reported in various models, the observed selectivity toward cancer cells could also stem from its impact on cancer stem‐like cells (CSCs), lysosomal function, and nutrient transport inhibition (Nagahara et al. 2005).

In our experimental models, GPE induces apoptotic death through caspase 3 activation, mitochondrial membrane integrity loss, and apoptotic morphological features such as cell shrinkage, chromatin condensation, micronuclei presence, and secondary necrosis. Mitochondrial dysfunctions, detected at TEM and after NAO staining, play a pivotal role in this network and may be an event further upstream of apoptosis, triggering the release of proapoptotic factors. Cardiolipin peroxidation affects the physiological functions of cell membranes, resulting in the loss of the inner membrane integrity and consequent release of cytochrome c, thereby promoting apoptotic death (Salucci et al. 2016).

GPE downregulates Beclin‐1, a critical protein for autophagy and autophagosome formation, which is often overexpressed in gastric cancers and linked to metastasis and poor prognosis (Ciechomska et al. 2009; Wang et al. 2015; Zhihong et al. 2013). This reduction suggests a tumor‐suppressor function, as high Beclin‐1 levels promote autophagy and reduce apoptosis. GPE may also interact with caspase‐3, a proapoptotic enzyme, disrupting Beclin‐1's autophagic activity and shifting the balance toward apoptosis (Luo and Rubinsztein 2010; Zheng et al. 2020). Despite reduced Beclin‐1 expression, GPE activates autophagy in AGS and KATO III cells. TEM shows degradative vacuoles digesting damaged membranes and organelles, confirmed by upregulated LC3B‐II, a key autophagosome protein (Hu and Reggiori 2022), suggesting that GPE‐induced autophagy is a damage response, ultimately leading to programmed cell death, as confirmed by ultrastructural and CLSM analyses. GPE shows antitumor effects by coordinating autophagy–apoptosis crosstalk, reducing Beclin‐1, increasing LC3B‐II, disrupting autophagic flux, and causing autophagosome accumulation, shifting autophagy toward cell death in GC cells.

## 5 Conclusions

The findings of this study suggest that GPE could be a promising nutraceutical candidate for gastric cancer prevention due to its selective cytotoxicity toward cancer cells and its ability to modulate multiple signaling pathways involved in tumor progression. Nevertheless, further investigations, particularly in vivo and clinical studies, must validate its efficacy and establish its safety profile. Additionally, its classification as a nutraceutical or functional food supplement within the European regulatory framework would require compliance with EFSA guidelines under Regulation (EC) No 1924/2006, necessitating robust scientific substantiation. If regulatory approval is pursued, a standardized characterization of GPE's active components and well‐structured human intervention trials will be necessary to support its health claim. Such an approach would also facilitate its integration into functional foods or dietary supplements to support gastric health. Moreover, the valorization of grape pomace through its transformation into a bioactive extract aligns with sustainability principles, offering a dual benefit of waste reduction and health promotion. Recent literature has confirmed the nutraceutical potential of grape pomace, not only as a source of polyphenols but also as a matrix rich in biologically active polysaccharides and antioxidants. For example, Atanacković Krstonošić et al. (Atanacković Krstonošić et al. 2025) optimized a surfactant‐assisted green extraction for maximizing phenolic yield from grape pomace using response surface methodology, underscoring the importance of process design in enhancing extract quality. Prata et al. (Prata et al. 2025) highlighted the multifunctional bioactivities of *Vitis vinifera* pomace‐derived nutrients, supporting their inclusion in nutraceutical formulations. Similarly, Garaigordobil et al. (Garaigordobil et al. 2025) demonstrated the potential of enzymatic degradation of cell wall components to recover valuable polysaccharides from both red and white pomace, broadening the range of usable bioactives. Clinically oriented studies have also begun validating the efficacy of grape pomace derivatives: Schiano et al. (Schiano et al. 2024) showed that a maltodextrinated GPE (MaGPE) significantly improved parameters in patients with diabetic retinopathy, while Annunziata et al. (Annunziata et al. 2021) and Zannella et al. (Zannella et al. 2023) reported cardioprotective, antioxidant, and antiviral effects of Taurisolo, a standardized polyphenolic extract from grape pomace, including benefits in neutrophil oxidative stress modulation and antiherpetic activity. These findings confirm the growing translational relevance of grape pomace in preventive health and support the rationale for its valorization within circular economy frameworks. The present study contributes to this expanding field by proposing a Verdicchio‐derived extract with selective activity against gastric cancer cells, thus combining nutraceutical innovation with sustainable resource reuse.

However, the potential benefits of this approach extend also to other areas such as environmental preservation.

Indeed, this work was developed within the principles of circular economy, repurposing grape pomace and highlighting the role of sustainable viticulture in reducing environmental impact through the transformation of winery by‐products into functional food ingredients. To substantiate this environmental benefit, we hypothesized that the greenhouse gas emissions would be avoided by reusing grape pomace for extract production instead of conventional disposal. Based on carbon balance models, the aerobic degradation of grape pomace may release approximately 0.9 t of CO_2_‐equivalent per ton of material. Although our study processed only 20 kg of pomace, this corresponds to an estimated saving of around 18 kg CO_2_ equivalent. While modest at laboratory scale, this impact becomes significant in industrial applications, confirming the environmental value of this valorization strategy. Further research is essential to fully explore GPE's nutraceutical potential and its role in advancing diet‐based cancer prevention and public health. Future studies should explore the clinical translation of GPE and its broader applications in sustainable health solutions.

## 6 Limitations and Future Prospects

The present study provides promising in vitro evidence of the selective anticancer activity of Verdicchio GPE against gastric cancer cells. However, several limitations must be acknowledged. All results were obtained in vitro; no in vivo or clinical investigations have been performed. Furthermore, while the extract was chemically characterized, a detailed assessment of the bioavailability and metabolism of the most active compounds remains to be addressed.

Future research will focus on validating the observed effects in animal models of gastric carcinogenesis and exploring the pharmacokinetics, tissue distribution, and safety profile of GPE after oral administration.

Additionally, future efforts will aim at scaling up the extraction process under food‐grade and GMP‐compliant conditions, ensuring reproducibility, stability, and regulatory compliance. This would enable the development of functional food products or dietary supplements containing standardized GPE. From a broader perspective, integrating grape pomace valorization into viticultural supply chains represents a relevant frontier in circular economy strategies. GPE could serve as a model for rethinking food waste as a source of bioactive compounds with applications in preventive health. Cross‐disciplinary collaborations between food scientists, pharmacologists, clinicians, and environmental experts will be key to advancing the translational potential of this sustainable approach.

## Author Contributions

**Matteo Micucci:** conceptualization (equal), investigation (equal), supervision (equal), writing – original draft (equal), writing – review and editing (equal). **Michela Battistelli:** conceptualization (equal), investigation (equal), validation (equal), writing – original draft (equal), writing – review and editing (equal). **Sabrina Burattini:** data curation (equal), writing – review and editing (equal). **Michele Mari:** data curation (equal), writing – review and editing (equal). **Michele Retini:** data curation (equal), writing – review and editing (equal). **Riham Osman:** investigation (equal), software (equal). **Ilaria Versari:** data curation (equal). **Anna Bartoletti Stella:** data curation (equal). **Federico Gianfanti:** data curation (equal). **Jude Okeke Udodinma:** data curation (equal). **Francesco Onesimo:** data curation (equal). **Serena Riela:** writing – original draft (equal), writing – review and editing (equal). **Matteo Canale:** data curation (equal). **Antonio Palumbo Piccionello:** writing – original draft (equal), writing – review and editing (equal). **Irene Faenza:** writing – original draft (equal), writing – review and editing (equal). **Sara Salucci:** data curation (equal), writing – original draft (equal), writing – review and editing (equal).

## Conflicts of Interest

The authors declare no conflicts of interest.

## Data Availability

Data will be made available upon request.
